# Prevention of lymphedema via axillary reverse mapping for arm lymph-node preservation following breast cancer surgery: a randomized controlled trial

**DOI:** 10.1186/s13037-019-0217-1

**Published:** 2019-11-14

**Authors:** Mohammed Faisal, Mohamed Gamal Sayed, Kerolos Antonious, Ahmmed Abo Bakr, Sherif Hussein Farag

**Affiliations:** Surgical Oncology Unit, Department of Surgery, Faculty of Medicine, Suez Canal University hospital, Circular Road, Ismailia, 411522 Egypt

**Keywords:** Breast cancer, Lymphedema, Blue dye, Axillary reverse mapping

## Abstract

**Background:**

Breast cancer, with an incidence of 32%, is the most frequent cancer among Egyptian women. The frequency of arm lymphedema after axillary surgery for breast cancer ranges from 7 to 77%. Axillary reverse mapping is a technique aimed to distinguish and conserve upper-limb lymphatics and lymph nodes during the course of axillary surgery and could help to prevent arm lymphedema.

**Methods:**

Patients (*n* = 48) were prepared for axillary lymph-node dissection. The study group and the control group each contained 24 individuals. In the study group, following dye injection, stained arm lymph nodes and lymphatics were conserved during axillary dissection, whereas control-group participants underwent the conventional procedure. All participants were re-evaluated after 6 months, and the incidence of lymphedema was recorded by measuring arm circumference at a level 10 cm proximal to the medial epicondyle. Arm lymphedema was defined as a change in the circumference of the ipsilateral upper extremity > 2 cm during the follow-up period.

**Results:**

Age, tumor size and N stage were not significantly different between the study and control groups. Lymph-node visualization was achieved in 20 participants (83.3%) in the study group. Suspicious stained lymph nodes were surgically removed from four individuals but showed no metastatic involvement. In 20 individuals in the study group, no stained lymph nodes were removed. The incidence of lymphedema in the control group was 16.7%, and the incidence in the study group was 4.2%.

**Conclusions:**

Axillary reverse mapping is a minimally invasive technique that can be performed during axillary lymph-node dissection, helping to prevent the subsequent development of arm lymphedema.

**Trial registration:**

#SCURCTN3276, retrospectively registered on 11 April 2017 at Research Ethics Committee at the Faculty of medicine-Suez Canal University.

## Background

Worldwide, ~ 1.67 million women are diagnosed with breast cancer every year. Breast cancer is second on the list of the most common cancers in the world and has the highest cancer incidence among the female population [1], including that in Egypt, where it represents 32.04% of female cancer incidence [2]. As the number of patients recovering from breast cancer treatment increases, the importance of limitation of the adverse effects of axillary surgery will also increase. One of these adverse effects is arm lymphedema, which has a frequency of 7–77% after axillary surgery [3].

Axillary reverse mapping (ARM) is a technique that has been devised based on the assumption that arm lymphedema results from disruption of arm lymphatics during axillary dissection. The axillary lymphatics and lymph nodes are anatomically linked to the lymphatic drainage of the arm; these nodes include the lateral or brachial group that lies below the axillary vein [4]. The purpose of ARM is to differentiate the lymphatics and lymph nodes of the arm from those of the breast during axillary surgery, to enable preservation of the arm lymphatics [5]. Currently available methods for ARM are based on injection and tracking of blue dye, fluorescent dye or a radioisotope, as reviewed elsewhere [6].

In the current study, our objectives were to assess the effectiveness of arm-node preservation by ARM for prevention of lymphedema in patients with breast cancer undergoing axillary lymph-node dissection (ALND), to determine the rate of arm-node involvement and to investigate the location of arm nodes. Our overall aim is to improve the quality of life of patients with breast cancer by reducing the adverse effects associated with axillary surgery.

## Methods

A randomized controlled trial was carried out in the Surgery Department of Suez Canal University Hospital and Ismailia Teaching Oncology Hospital in Egypt from June 2017 to January 2018. The study design was reviewed by the Research Ethics Committee at the Faculty of medicine-Suez Canal University at its meeting on 11/04/2017 with reference number (#3276). The study adhered to CONSORT guidelines.

The following formula was used to calculate the required sample size:

*n* = (Zα/2 + Zβ)2 * (p1(1 − p1) + p2(1 − p2))/(p1 − p2)2,

where *n* is the sample size of each group (with a 1:1 ratio of group sizes), α is the probability of type I error (set at 0.01), Zα/2 is the critical value of the normal distribution at α/2, β is the probability of type II error (set at 0.2), Zβ is the critical value of the normal distribution at β (for β = 0.2, Zβ = 0.84), and p1 and p2 are the expected sample proportions (incidences) in the two groups. Yue et al. previously demonstrated incidences of lymphedema of 33.1% with ARM and 5.9% without ARM [7]. These values were used for p1 and p2, resulting in a calculation of *n* = 24 for each group in our study. A total of 48 patients diagnosed with breast cancer were investigated.

ALND was indicated for all participants, who were randomly assigned (via a sequence generated in Microsoft Excel) in a 1:1 ratio to either the study or control groups. The technique was performed by a single surgeon, who was given the randomly generated treatment allocations within sealed, opaque envelopes. When a patient consented to enter the trial, the envelope was opened, and the patient underwent the allocated surgery.

**Fig. 1:**
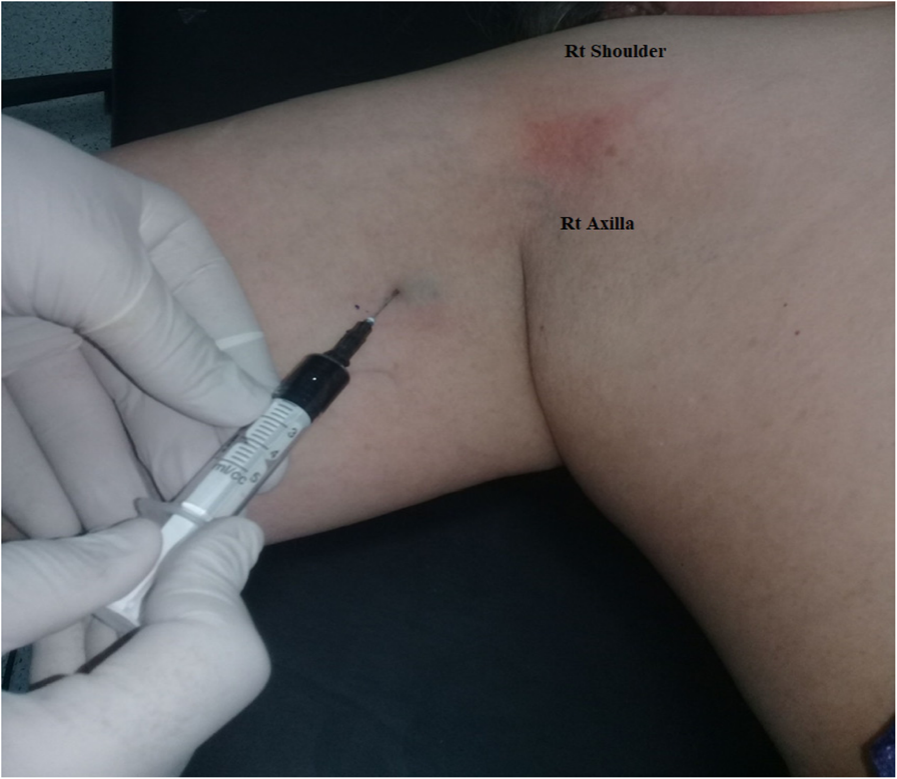
Injection of blue dye

In the study group, general anesthesia was employed then ~ 2.5 ml of 1% (w/v) methylene blue dye was injected subcutaneously into the medial intramuscular crease of the upper arm on the same side (Fig. 1**)**. The injection site was massaged, and the arm was elevated for a few minutes to aid dye migration toward the axilla. Surgery for breast cancer, either modified radical mastectomy or wide local excision of the lesion, was then performed, followed by axillary dissection (~ 20 min after dye injection).

**Fig. 2:**
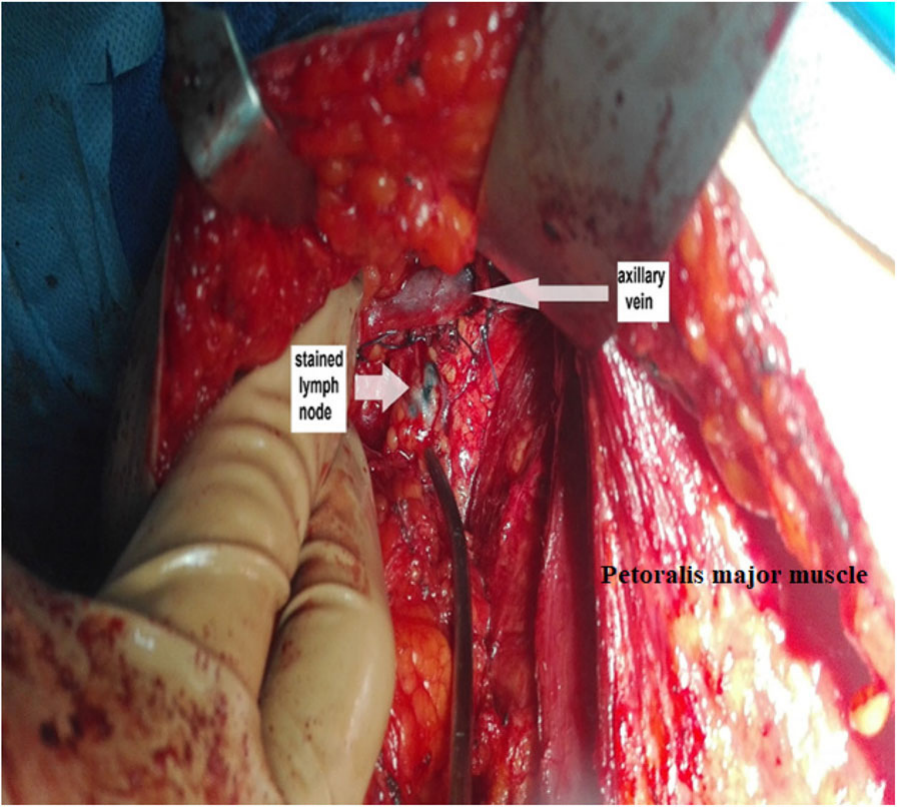
Identification of a stained lymph node below the axillary vein

**Fig. 3:**
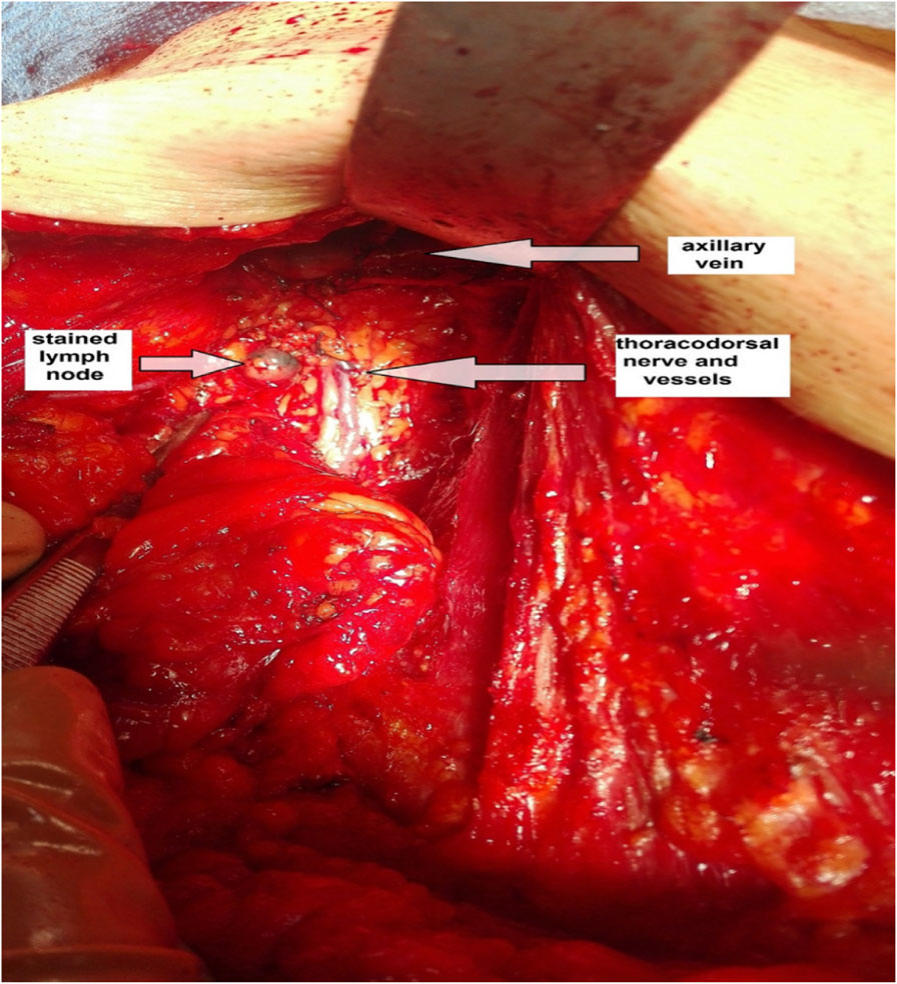
Location of a stained lymph node lateral to the thoracodorsal trunk 2 cm below the axillary vein

Lymphatic arm drainage (LAD) was identified by observation of stained lymphatics and stained lymph nodes draining the arm (within the lateral compartment of the axilla) (Fig. 2) that were preserved as a part of the ARM procedure during ALND (Fig. 3). Any variations in stained arm lymphatics associated with the site and their sizes were noted.

**Fig. 4:**
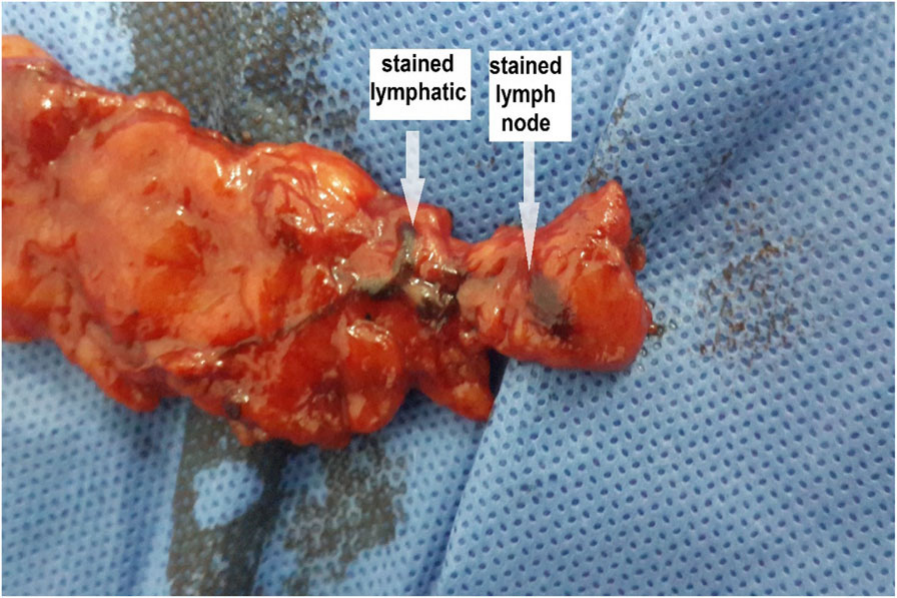
Example of axillary lymph-node dissection. For this patient, the total number of excised lymph nodes was 19. Three of these nodes were stained, and there was no evidence of metastasis in the stained nodes. Among the 16 unstained nodes, metastasis was detected in five nodes

Removal of stained lymph nodes was carried out only in patients with suspicious lymph nodes (Fig. 4), which were identified by the following criteria: 1) multiple suspicious or amalgamated lymph nodes in the axilla; 2) enlarged node (> 1 cm); 3) firm or hard node; and 4) anatomical location other than lateral or above the thoracodorsal pedicle.

In the control group, classic axillary dissection was performed without conservation of the upper-limb lymphatics and lymph nodes.

All patients were revaluated after 6 months, and the incidence of lymphedema was recorded.

### Arm circumference measurement

Arm circumference was measured at a level 10 cm proximal to the medial epicondyle before surgery and 6 months after surgery. Changes between arm circumference of the ipsilateral and contralateral upper extremity in each group were compared. In the ipsilateral upper extremity, arm circumferences before surgery and 6 months after surgery were also compared. Arm lymphedema was defined as a change in the circumference of the ipsilateral upper extremity > 2 cm during the follow-up period [8].

## Results

The mean age of the study population was 52 ± 11 years. All participants had invasive ductal carcinoma, as shown by Tru-cut biopsy (Table 1); 22.9% had T1 lesions, 56.3% had T2 lesions, and 20.8% had T3 lesions. Pathologic axillary lymph-node staging demonstrated that 29.3% of participants had pN0 disease, 35.4% had pN1 disease, 18.8% had pN2 disease, and 16.7% had pN3 disease. Regarding the type of surgery, 27.1% of participants underwent conservative breast surgery (wide local excision of the lesion), whereas 72.9% underwent modified radical mastectomy.

**Table 1:** Histopathological tumor characteristics of the study population

Variable	Parameter	Control group, *n* (%)	Study group, *n* (%)	Total, *n* (%)
Histopathology	IDC	24 (100)	24 (100)	48 (100)
T stage	T1	9 (37.5)	2 (8.3)	11 (22.9)
	T2	9 (37.5)	18 (75)	27 (56.3)
	T3	6 (25)	4 (16.7)	10 (20.8)
N stage	N0	7 (29.2)	7 (29.2)	14 (29.2)
	N1	13 (54.2)	4 (16.7)	17 (35.4)
	N2	2 (8.3)	7 (29.2)	9 (18.8)
	N3	2 (8.3)	6 (25)	8 (16.7)

*IDC* invasive ductal carcinoma

Out of 24 participants with ARM, stained lymphatics were visualized in 18 (75.0%), and stained lymph nodes were visualized in 20 (83.3%) (Table 2). Of the stained nodes, 95.9% were situated between the second intercostobrachial nerve and the lower limit of the axillary vein. In four participants, some stained lymphatics and nodes (mean 2.25, range 1–3 lymph nodes per person) were removed and assessed by histopathological examination. Metastasis was not identified in any of the removed stained lymph nodes (Table 3). Arm lymphedema was reported in one individual in the study group (4.2%), and four in the control group (16.7%) (Table 4).

**Table 2:** Characteristics of axillary lymph-node dissection surgery in the study population

Variable	Parameter	Control group, *n* (%)	Study group, *n* (%)	Total, *n* (%)	χ^2^	*p*-value
Type of breast cancer surgery	Conservative	8 (33.3)	5 (20.8)	13 (27.1)	0.949	0.330
	MRM	16 (66.7)	19 (79.2)	35 (72.9)		
Visualization of lymphatics	Yes	N/A	18 (75)	N/A	N/A	N/A
	No	N/A	6 (25)	N/A		
Visualization of lymph nodes	Yes	N/A	20 (83.3)	N/A	N/A	N/A
	No	N/A	4 (16.7)	N/A		

*MRM* modified radical mastectomy; *N/A* not applicable

**Table 3:** Histopathological status of surgically removed stained lymph nodes from four patients in the study group

Arbitrary case number	N stage	Total number of nodes removed	Total number of metastatic nodes removed	Number of stained nodes removed	Number of metastatic stained nodes removed
1	N3	15	13	1	0
2	N2	19	5	3	0
3	N1	13	2	3	0
4	N1	14	1	2	0

**Table 4:** Lymphedema incidence and distribution during 6 months follow-up in the study population

Variable	Parameter	Control group, *n* (%)	Study group, *n* (%)	Total, *n* (%)	χ^2^	*p*-value
Incidence of lymphedema	Yes	4 (16.7)	1 (4.2)	5 (10.4)	2.009	0.156
	No	20 (83.3)	23 (95.8)	43 (89.6)		

## Discussion

ALND is the main method for final staging of breast cancer [9]. The morbidities associated with ALND, especially upper-limb lymphedema, lead to reductions in quality of life, because affected individuals suffer from disfigurement, pain, numbness, restriction of movement and recurrent infections [3].

The ARM technique was developed on the basis of evidence of the existence of separate lymphatic pathways for the arm and the breast. This evidence suggests that upper-limb lymphedema results from disruption during axillary dissection of the lymphatic channels and lymph nodes associated with arm lymph drainage. Identification and preservation of these lymphatics and lymph nodes should therefore limit the incidence of morbidities associated with ALND [5, 10].

For ARM in this study, the anatomical location of dye injection in the upper part of the arm along the medial intermuscular crease was selected because it is the site where nearly all arm lymphatics aggregate, and injection in this area enables rapid migration of the dye to the axilla [8]. Moreover, this site is preferred because it conceals the blue mark resulting from injection, which may remain for up to 6 months [11].

The effectiveness of ARM technique is described by its value in identifying arm nodes and reducing arm lymphedema also by the safety of the technique. In this study stained lymphatics were visualized in 18 individuals (75.0% of the study group), and stained lymph nodes were documented in 20 individuals (83.3%). Only two participants had no visualization of stained lymph nodes or lymphatics in the axilla; the axillary dissection showed multiple amalgamated nodes, which made visualization of the blue dye difficult. In a previous study conducted by Nos et al. [12], the LAD mapping rate was 91%, with positive identification in 21 of 23 individuals, with an ARM technique combining injections of radioactive isotope and blue dye. In a larger study, Tummel et al. [13] identified stained arm nodes and lymphatics in 153 of 213 individuals (71.8%) who underwent ARM.

In our study, 95.9% of the stained nodes were located between the lower aspect of the axillary vein and the second intercostobrachial nerve lying lateral to the thoracodorsal nerve and vessels. Only one patient (4.1%) had stained nodes that were situated medial to the thoracodorsal nerve and vessels. These results are similar to those of a previous study [8] in which stained lymph nodes inferior to the axillary vein and above the second intercostobrachial were found in 97% of the study population. Clough et al. [14] identified stained arm nodes 1 cm inferior to the axillary vein, lateral to the thoracodorsal nerve and vessels. Kumar et al. [15] found no metastatic involvement in stained arm nodes located lateral to the thoracodorsal nerve and vessels.

In our study, the mean number of axillary nodes removed during ALND was 13.4 per person in the study group and 17.4 in the control group; the difference between these means was statistically significant. Although the quantity of retrieved lymph nodes in the ARM group was less than the number of resected nodes with classic axillary dissection, there was no statistically significant difference between the number of harvested nodes and the number of nodes that were positive for malignancy. Notably, the mean number of excised nodes in our ARM study group was greater than the 10 nodes previously suggested to be the minimum number resected for axillary clearance to be oncologically successful [16].

In four of the individuals in our study group, stained lymph nodes were classified as suspicious by virtue of being firm and > 1 cm in diameter or being located medial to the thoracodorsal nerve and vessels. These suspicious nodes were surgically removed and separately assessed by histopathological examination; the mean number of removed blue lymph nodes was 2.25, ranging from 1 to 3 nodes. None of the removed stained nodes showed any metastatic spread.

In our study, we relied on the variation in arm circumference, as previously defined [8], to determine the occurrence of arm lymphedema during the follow-up period. Chirag and his colleagues [17] have also indicated that self-assessed symptoms of discomfort, numbness and pain may be important for assessment of the incidence of lymphedema.

In our study, the 4.2% incidence of lymphedema in the study group was not significantly different from the 16.7% incidence in the control group. This lack of significance may be the result of the small number of patients included in our study. Tausch et al. [18] studied 143 patients with breast cancer, with preservation of arm lymphatic drainage in 52.7% of the cohort. Although the incidence of lymphedema in that study was 43% in the group without preservation and only 23% in the ARM group, as in our study there was no significant difference in incidence between the groups [18]. However, Yue and colleagues enrolled 265 patients with breast cancer and identified a significant difference in the incidence of lymphedema (33.7% in the control group versus 5.93% in the ARM group; *P* < 0.001) [7].

The methylene blue dye that is used for staining of lymph nodes is safe for subcutaneous injection, with only rare reports of allergic reactions. One drawback of the use of this dye, however, is skin tattooing, which may last from 1 week to 6 months. To counter this drawback, the inner aspect of the upper arm can be chosen as the injection site to help mask the tattooed skin [19, 20].

### Study limitations

One limitation of our study was the small sample size, which may have prevented us from demonstrating a significant difference in lymphedema incidence between the groups; large-scale studies are strongly recommended to validate the results. Another limitation was that the short-term follow-up of 6 months may not have been long enough to distinguish transient lymphedema resulting from acute surgical edema from permanent lymphedema, suggesting that longer follow-up is needed.

## Conclusions

ARM is a minimally invasive technique that can be readily added to ALND and that can help prevent arm lymphedema. The use of ARM for LAD mapping and avoidance of excision of arm lymphatics and nodes was associated with a lower incidence of arm lymphedema than classic ALND surgery in our study population. However, we recommend implementing future studies on the ARM procedure in a larger number of patients, to obtain statistically significant results.

## Data Availability

The datasets used and/or analyzed during the current study are available from the corresponding author on reasonable request. All data generated or analyzed during this study are included in this published article [and its supplementary information files].

## Abbreviations

ARM: Axillary reverse mapping ALND Axillary lymph-node dissection LAD Lymphatic arm drainage
